# Data on coronary artery calcium score performance and cardiovascular risk reclassification across gender and ethnicities

**DOI:** 10.1016/j.dib.2016.01.002

**Published:** 2016-01-13

**Authors:** Marat Fudim, Sandip Zalawadiya, Devin K. Patel, Ugochukwu O. Egolum, Luis Afonso

**Affiliations:** a Department of Internal Medicine, Division of Cardiology, Duke University Hospital, Durham, NC, United States; b Department of Internal Medicine, Vanderbilt University Hospital, Nashville, TN, United States; c Division of Cardiology, Vanderbilt University Hospital, Nashville, TN, United States; d Department of Internal Medicine, Division of Cardiology, Wayne State University, Detroit, MI, United States

**Keywords:** Risk stratification, Coronary calcium scoring, Gender, Ethnicity, MESA, {C}{C}

## Abstract

The current guidelines recommend the new risk score, Atherosclerotic Cardiovascular Disease score (ASCVD), to assess an individual׳s risk of future cardiovascular disease (CVD) events. No data exist on the predictive utility of ASCVD score with the incremental value of coronary artery calcium scoring (CACS) across ethnicities and gender. Multi-Ethnic Study of Atherosclerosis (MESA) is a population based study (*n*=6814) of White (38%), Black (28%), Chinese (22%) and Hispanic (12%) subjects, aged 45–84 years, free from clinical cardiovascular disease. We performed a post-hoc analysis of 6742 participants (mean age 62, 53% female) from the MESA cohort. We evaluated the predictive accuracy for the ASCVD score for each participant in accord with the American College of Cardiology/American Heart Association guidelines using pooled cohort equations. Similar to the publication by Fudim et al. “The Metabolic Syndrome, Coronary Artery Calcium Score and Cardiovascular Risk Reclassification” [1] the analytic properties of models incorporating the ASCVD score with and without CACS were compared for cardiovascular disease CVD prediction. Here the analysis focused on ASCVD score (with and without CACS) performance across gender and ethnicities.

**Specifications Table**

**Table:** 

Subject area	*Medicine*
More specific subject area	*Cardiology*
Type of data	*Tables*
How data was acquired	*MESA cohort data*
Data format	*Analyzed*
Experimental factors	*Demographic stratification*
Experimental features	*Post-hoc analysis of limited access dataset of MESA study*
Data source location	*Washington, USA*
Data accessibility	*Data is within this article.*

## Value of the data

• Our analysis should be evaluated in other contemporary cohorts to confirm our findings.
• Our data invites a more in depth analysis (quantitative and qualitative) of the utility of CACS to reclassify risk.
• The value of CACS to reclassify risk should be compared to other “biomarkers” using the new ASCVD score.
• The value of CACS should be explored in other subgroups like different age groups and renal disease.

## 1. Data

We performed post-hoc analysis of limited access dataset of MESA study obtained from the National Heart, Lung and Blood Institute (NHLBI). The publically available dataset is current through Exam 4 with a median follow-up time of 7.5 years. The sample size includes a total of 6742 participants. ASCVD score was calculated for each participant based on the pooled risk estimation equation recommended by 2013 ACC/AHA Guideline on the Assessment of Cardiovascular Risk [2]. As suggested by the guidelines, Hispanic Americans and Asian Americans were applied the risk estimates of non-Hispanic Caucasian American participants. The outcome of interest was hard CVD (CVDh) events, which included participants with myocardial infarction, death due to myocardial infarction, resuscitated cardiac arrest, stroke and death from stroke. A comparable analysis of this data set focusing on the metabolic syndrome is already published [1].

## 2. Experimental design, materials and methods

Baseline characteristics were computed and compared for each gender and race using chi-square test for categorical variables (presented as %) and one-way ANOVA test or *t*-test for continuous variables as deemed appropriate. CACS was transformed as log of CACS +1 when analyzed as a continuous variable.

We first evaluated predictive accuracy of ASCVD as a standalone test for each gender and race through multivariable Cox proportional hazard analysis. Harrell׳s C statistics were determined to compare model discrimination in a time-dependent manner. Subsequently, CACS was evaluated as an adjuvant to the base models of ASCVD score with Harrell׳s c-statistics calculated and compared for each demographic. Likelihood Ratio test (LR test, -2log likelihood ratio test) and Bayesian information criterion, which provides information about the probability that a given independent variable is a part of the true model, were analyzed to assess the global fit of the models.

As published scoring systems estimate 10-year risk, risk estimates were recalibrated for 7 years follow up to allow for comparison within the observed follow-up period [3], [4], [5], [6]. Recalibrated risk categories were as following: Low risk <5.25% and high-risk ≥5.25%. Risk reclassification was then assessed using previously published methods of calculating categorical [7] NRI, continuous NRI [8] (a measure ranging from −2 to 2 used to assess improvement of a model׳s ability to predict outcomes with addition of a separate risk factor) and integrated discrimination improvement (IDI, a measure of difference in discrimination slopes of events and nonevents between two risk prediction models) [7].

All the statistical analyses were performed using STATA 10.0 (StataCorp, College Station, Texas), SPSS 20 (IBM Corp, Armonk, New York) and statistical programming language R 3.1.1(R Foundation, Vienna, Austria). Table. 1

## Figures and Tables

**Table 1:** Analytic properties of ASCVD score* with and without the coronary artery calcium score across genders and ethnicities for prediction of cardiovascular disease events ©.

**Demographic**	**Male**	**Female**	**Caucasian**	**Chinese American**	**African American**	**Hispanic**
Number of patients/ Events	3186/165	3556/131	2599/140	801/20	1850/92	1492/74
Event rate [per 1000 person-years]	6.1%	3.7%	5.4%	2.5%	5.0%	5.0%
***Discrimination***						
C Statistic for ASCVD	0.705	0.766	0.734	0.734	0.707	0.800
C Statistic for ASCVD+CACS	0.730	0.784	0.753	0.747	0.740	0.809
Improvement in C Statistic (*p* value)	0.025 (*p*=0.047)	0.018 (*p*=0.19)	0.019 (*p*=0.18)	0.013 (*p*=0.66)	0.033 (*p*=0.11)	0.009 (*p*=0.45)
***Calibration***						
Hosmer–Lemeshow Chi-square (*p* value)	8.587 (*p*=0.38)	16.715 (*p*=0.033)	11.9 (*p*=0.16)	4.9 (*p*=0.77)	11.0 (*p*=0.20)	12.3 (*p*=0.14)
Bayes information criterion support for model with CACS	Very strong	Very strong	Very strong	Positive	Very strong	Very strong
***Reclassification***						
Categorical NRI (*p* value)	0.080 (*p*=0.037)	0.095 (*p*=0.039)	0.111 (*p*=0.02)	−0.121 (*p*=0.11)	0.111 (*p*=0.082)	0.024 (*p*=0.61)
Category-less NRI (*p* value)	0.437 (*p*<0.001)	0.488 (*p*<0.001)	0.587 (*p*<0.001)	0.701 (*p*=0.003)	0.500 (*p*<0.001)	0.472 (*p*<0.001)
Integrated Discrimination Index (*p* value)	0.0117 (*p*<0.001)	0.0069 (*p*=0.032)	0.012 (*p*<0.001)	0.005 (*p*=0.27)	0.014 (*p*<0.001)	0.006 (*p*=0.23)

*Abbreviations***:** ASCVD=Atherosclerotic cardiovascular disease, CACS=Coronary artery calcium score; NRI=Net reclassification index.

* ASCVD risk score was calculated in compliance with pooled cohort equation provided by American College of Cardiology/ American Heart Association guidelines which incorporated gender and ethnicity based risk estimated incorporating following risk factors: age, total cholesterol, high density lipoprotein cholesterol, systolic blood pressure, treatment for hypertension, diabetes, current smoking. For Chinese and Hispanic Americans, risk estimates of Caucasian Americans were assigned as suggested by the guidelines.

© CVD events included myocardial infarction, death due to myocardial infarction, resuscitated cardiac arrest, stroke, and death from stroke.In calculation of categorical NRI, risk categories for ASCVD score were re-calibrated to provide corresponding risk estimates for 7 years follow-up.The *interaction* between gender and ASCVD for prediction of Hard CVD was not statistically significant (*p*=0.19).
